# Rational Prediction with Molecular Dynamics for Hit Identification

**DOI:** 10.2174/156802612804910313

**Published:** 2012-09

**Authors:** Sara E Nichols, Robert V Swift, Rommie E Amaro

**Affiliations:** 1 University of California, San Diego Department of Pharmacology; 2 University of California, San Diego Department of Chemistry and Biochemistry; 3 Howard Hughes Medical Institute

**Keywords:** Computational methods, docking, drug design, flexibility, molecular dynamics, structure-based prediction, validation, statistical performance analysis.

## Abstract

Although the motions of proteins are fundamental for their function, for pragmatic reasons, the consideration of protein elasticity has traditionally been neglected in drug discovery and design. This review details protein motion, its relevance to biomolecular interactions and how it can be sampled using molecular dynamics simulations. Within this context, two major areas of research in structure-based prediction that can benefit from considering protein flexibility, binding site detection and molecular docking, are discussed. Basic classification metrics and statistical analysis techniques, which can facilitate performance analysis, are also reviewed. With hardware and software advances, molecular dynamics in combination with traditional structure-based prediction methods can potentially reduce the time and costs involved in the hit identification pipeline.

## INTRODUCTION

Proteins are flexible macromolecular structures that can undergo a wide range of motions considered fundamental for their function. Movement can be subtle, including bond vibrations and side chain reorientation. Larger motion is also typical and includes hinge-like rigid domain translations and rotations, backbone rearrangements that may result in alternative secondary structure and loop region flexibility. Flexibility can also entail an equilibrium distribution among iso-energetic conformations that are intrinsically disordered. This hierarchy of motion is relevant in structure based discovery and design. For example, many known proteins have been co-crystallized in alternative conformations, distinct from the unbound crystallized state. This implies motion is fundamental to the co-crystallized complex, and suggests alternative conformational states may exist that are relevant, and imperative, to the discovery of other potential ligands. While historically, protein flexibility has been largely ignored in high-throughput methods used in drug discovery and design, recent software and hardware advances have allowed for the consideration of implicit or explicit protein motion [1-6]. 

Ideally, the use of computational methods should reduce the cost and time involved in structure-based drug discovery. We highlight here the general concepts behind protein motion and its relation to molecular interactions. Two major types of predictions are discussed including binding site classification, and virtual screening. During hit discovery, prediction, carried out in a high-throughput fashion, assists practically in the process of research and discovery. Methods should be carefully evaluated on a system-dependent basis, and quantfiable validation should be established at the onset to enable the user to critically decide between several methods and convey the results to potential collaborators with confidence. This review discusses these concepts and how molecular dynamics conformational diversity can enhance the structure-based drug design process. 

## MOLECULAR FLEXIBILITY AND ITS ROLE IN DRUG DISCOVERY AND DESIGN

Protein motion is key to functional mechanisms that require biomolecular association, such as catalysis, chaperone-assisted folding, protein-protein and protein-nucleic acid interactions, and allosteric regulation. Ligand binding is designed to enhance or interrupt these functions, and therefore may be tightly coupled to protein motion. Ligand association can be as simple as substrate binding prior to enzyme turnover, or as complex as the signal transduction cascades that orchestrate life on the cellular level. It has been shown that most ligands are buried upon association with a protein, suggesting fundamental rearrangement of the receptor is required for complexation [7]. These motions can involve a single side chain movement, such as the repositioning of a catalytic residue in an enzyme, or a coordinated isomerization that occurs concertedly among a group of residues. The timescales for relevant biophysical phenomena cover a wide range, from femtosecond bond vibrations to collective motions requiring seconds to occur. Methyl rotation, loop motion and side-chain rotamer relaxation all happen on the picosecond to microsecond time scale [8]. Frequently, there is a compensation of enthalpy changes, or changes of the average internal energy of a system, and entropy changes, or changes in the measure of favorable disorder of a system. For example, one might observe a favorable decrease in enthalpy upon binding, as strong hydrogen bonds form between the ligand and receptor. This offsets the entropic penalty that results from rotational and translational ligand confinement. Often enthalpy is the primary consideration in potency optimization, but entropy can also be factored into the strategy [9]. Furthermore, the free energy of the water at the binding site may also play an important, even active role in molecular association [10]. Flexibility-function studies can lead to novel design strategies, and broaden the idea of drug action, through correlated networks of residues [11].

Flexible degrees of freedom, in addition to titratable side chains and non-bonded coulombic and dispersive interactions, produce a rugged, multidimensional energy landscape. The relative populations of the energy wells along this landscape define the thermodynamic properties of the system, while the transitions between them yield the system’s kinetics. Both thermodynamics and kinetics are fundamentally important considerations to understand proteins in motion. Developing efficient sampling methods for understanding minima and the transitions between them are current active areas of research [12]. This multidimensional complexity is a challenge for computational modeling with respect to drug discovery and development. Often, an approximate, rapid, high-throughput method is more appropriate for fast-paced pharmaceutical settings than a potentially more accurate, but time-consuming, technique. Among current theories of molecular association, two main concepts prevail, namely, induced fit and conformational selection. Induced fit theory assumes that the ligand influences the shape the protein receptor takes upon binding. Conformational selection hypothesizes that the bound-state conformation exists in the apo protein ensemble, and that upon ligand binding, the population shifts such that the bound-state conformation becomes the most populated conformer [13, 14]. For different targets, these two theories are not necessarily mutually exclusive and can be considered simultaneously, sequentially, or independently [15, 16].

Protein motion can be quantified with a number of experimental techniques. These include hydrogen deuterium exchange, Förster and bioluminescence energy transfer (FRET/BRET), nuclear magnetic resonance, solution X-ray scattering, circular dichroism, electron microscopy, and X-ray crystallography. One classic scenario from early crystallographic studies, that implies the requirement of protein flexibility in ligand association, is the experimentally observed conformations of the heme-based oxygen binding proteins, myoglobin and hemoglobin. Space-filled models of these proteins revealed no clear oxygen binding pathway to the heme [17]. It was clear that thermal fluctuations were necessary to facilitate the ligand binding process. Other experimental methods demonstrate the relationship between conformational reorganization and biomolecular function. A second, more contemporary, example is the use of NMR to classify dynamics relevant for adenylate kinase catalysis. Kern and co-workers proposed a linkage between picosecond to nanosecond motions and larger amplitude domain rearrangements that are important in facilitating catalytic activity [8]. Observations such as these inextricably link motion to function, and should be exploited for discovery and design purposes. 

## PREDICTION OF MOLECULAR RECOGNITION SITES

Molecular recognition site detection can be used early on in the pipeline of structure-based drug design (SBDD) to identify where a drug could possibly bind. Here we refer to three of the most commonly targeted types of binding sites, active sites, allosteric sites and protein-protein interaction sites. Most drugs are discovered or designed to bind the known active site, where an enzymatic reaction happens. Allosteric sites, distinct from the active site, are where a molecule binds and influences receptor activity and can also be targeted. These types of interactions have the advantage that they are often not conserved, and therefore can be more unique, or selective for the desired target, circumventing off-target interactions. Finally small molecules can be designed to target protein-protein interfaces, to prevent protein association into functional complexes. Due to crystallographic conditions, a binding site on a protein may at some point exist in an alternative shape, extend to interact with crystallographically undetected residues, or be completely distinct from a known binding site where a small molecule has already been observed. 

Binding pockets are characterized by shape and complementarily to prospective binders, but traditionally would include detection of active and allosteric sites. The nature of a binding site is constantly in fluctuation, including the contour, the side-chain dihedral angles, and potential polar and charged contacts. High affinity and specificity is a careful balance between opposing forces and interactions. There are a variety of different binding site detection methods. Broadly, these can be characterized into three main classification algorithms: geometric methods based on the surface of the site, energetic methods based on physical and chemical properties of the binding site, and knowledge-based methods that incorporate data gathered from other sources besides the protein structure [18-20]; methods that fall into these groups have been recently reviewed [19]. 

Defining what constitutes a binding site is problematic, particularly for the detection of druggable pockets in protein-protein interaction sites. Protein-protein interaction sites, tend to be shallow, hydrophobic, and extended surfaces [21]. Clackson and Wells defined hot spots as residues at a protein-protein interaction (PPI) site that are essential for association, and dominate the binding free energy [22]. These hot spots can be utilized as starting points for molecular docking and design optimization protocols. [23-25]. Computational prediction methods for these crucial areas have also been recently reviewed [26]. While targeting PPIs with small molecule inhibitors has been challenging, the concept of identifying epitopes important for binding is relevantly extended to other types of molecular recognition where clear binding cavities are not obvious, such as nucleic acid-protein or lipid-protein binding sites.

## SMALL MOLECULE DOCKING AND VIRTUAL SCREENING

Docking and virtual screening can be used to identify potential small molecules binders, and most docking programs reduce the search space and computational time by requiring the user to specify the putative binding site. Small molecule docking is the process of computationally fitting a ligand into a biomolecule, typically held rigid in a single conformation. To expedite the required calculations, early docking programs also fixed the ligand conformations and often relied on steric complementarities to rank the plausibility of the predicted compound orientations [27]. As Moore’s law [28] evolved and processor speed increased exponentially, ligand flexibility was introduced [29], and force fields were used to provide a classical estimate of ligand-receptor interactions. Flexibility allowed the ligand to relax in response to the binding pocket requirements, which were still held rigid, while the force field guided physically reasonable interactions, such as hydrogen bonds. Today, these force field descriptions are generally known as “scoring functions” [30, 31] and may be augmented by a variety of energetic terms, such as solvation and entropy; these typically provide a rough estimate of binding affinity. To further speed the calculations and facilitate the rapid docking of large compound databases, scoring function interactions can be pre-calculated and stored on grids for speedy look up [31-33]. Many docking programs are grid-based, and current state-of-the-art methods are very fast, which allows routine screening of large compound databases [34]. Moreover, the growth in docking technology has resulted in a large number of high-quality, freely available docking programs. Many of these programs can be found at click2drug.com, an excellent compendium resource of valuable *in silico* drug design tools.

Docking is an important tool in contemporary lead discovery. Its value lies in the potential to improve the efficiency and reduce the costs of modern high throughput screens (HTS) and ultra high throughput screens (uHTS) during hit discovery. Since its initial development in the early to mid 1990’s, modern HTS has grown to dominate lead discovery efforts and has undergone an increasing trend toward miniaturization [35]. Miniaturization resulted in smaller well volumes and a larger number of wells on each assay plate. Still, the cost of reagents and other consumables can be significant, particularly when large corporate or commercial databases, with compounds numbering in the millions, are considered [35]. To reduce costs, rather than randomly screening an entire database, docking can be used to rationally prioritize a subset for testing. This prioritization process is known as virtual high throughput screening (vHTS). In a typical vHTS, database compounds are docked into a single crystal structure and ranked according to their predicted binding affinities. Ideally, all of the binders would be ranked ahead of all of the non-binders, tremendously reducing experimental HTS expenditures. In practice, these results are never achieved, and even carefully validated docking protocols may fail to identify novel hits.[36, 37] Nevertheless, vHTS has demonstrated success across a range of targets, from dengue virus protease [38], to HIV-1 reverse transcriptase[39] to the RNA editing ligase from *Trypanosma brucei*, the causative agent of African sleeping sickness [40, 41], among others [34]. Despite successes, efforts to improve vHTS performance, including ensemble-based methods [42-44], are a vibrant area of research today.

## PREDICTION AND PERFORMANCE EVALUATION

As virtual screening method performance can vary from system to system, a sound, rigorous method of evaluation is important when developing a protocol. While we focus on virtual screening examples here, the concepts introduced are general and apply to other classification problems that occur in SBDD. Regardless of the classification method, we want to employ the very best protocol at our disposal to ensure project success. In the case of a virtual screen, this means that the list of compounds we recommend for testing contains as many true binders as possible. In the next section, we introduce the Receiver Operating Characteristic (ROC) curve [45] and the area under the curve, or AUC, as classification metrics and gauges of virtual screening performance. We also discuss the origins and statistical characterization of performance variability.

### ROCing Performance

Numerous virtual screening performance metrics have been published in the literature and have been discussed at length [46, 47]. Some popular examples include the enrichment factor EF, robust initial enhancement (RIE) [48], and the Boltzmann-Enhanced Discrimination of ROC (BEDROC) [46]. However, the AUC of a ROC curve is perhaps the most frequently applied and has been championed as *the* virtual screening performance metric [49, 50]. Below, we characterize the properties of ROC curves, and their corresponding AUC values, through several numerical illustrations. 

For the purposes of assessment, virtual screening is a binary classification problem [45], and the ROC curve is an easily interpreted graphical representation of docking protocol performance. During a virtual screen the estimated binding affinities, or binding free energies, can be used to rank compounds. Compounds with more favorable binding affinities will receive a better rank and are more likely to be recommended for subsequent experimental validation. To understand how a ROC curve is constructed, and why it reflects docking performance, it is useful to plot the distribution of predicted binding affinities for both the binders and non-binders. For example, Fig. (**1A**) represents a hypothetical virtual screening experiment in which the predicted distributions of the binders and non-binders are represented by black and gray curves, respectively. These normal distributions were constructed to have means of -5 and -3 kcal/mol, respectively, and identical standard deviations of 2 kcal/mol. A binding affinity threshold can be chosen such that all of the compounds with higher binding affinities will be tested. For example, the vertical black dashed line represents a -5 kcal/mol threshold. All of the compounds to the left of the dashed black line will be tested and all of the compounds to the right will not. Since the compounds represented by the black curve are binders, integrating from negative infinity up to the threshold gives the fraction of the binders we’d expect to discover if we chose a -5 kcal/mol threshold. These are the “true positives,” *i.e.* the compounds predicted to bind that actually bind. The value of the integral is often called the “true positive rate,” or TPR. On the other hand, if the gray curve is integrated from negative infinity up to the threshold, the fraction of non-binders we’d expect to recommend for experimental validation is given. These compounds are “false positives.” That is, the compounds that were predicted to bind that actually do not. Similarly, the value of this integral is often called the “false positive rate,” or FPR. By repeating the integration process while continuously varying the threshold, the true and false positive rates can be determined as functions of the threshold value Fig. (**1B**). In the language of probability theory and statistics, these curves are called cumulative distribution functions. Plotting the TPR as a function of the FPR yields a ROC curve Fig. (**1C**). This information is useful, as docking protocols that yield higher TPRs at a fixed FPR are expected to produce a greater number of actives at a given threshold. Indeed, the TPR at FPR of 0.5%, 1%, 2%, and 5% have been suggested as standard measure of virtual screening enrichment [49]. 

The area under the ROC curve is a valuable metric of virtual screening performance. To understand why, it is again useful to consider the distribution of predicted binding affinities, their corresponding cumulative distribution functions, and ROC curves. If a docking protocol has no discriminatory ability, then the distribution of predicted binding affinities, as well as their cumulative distribution functions, will be identical for the binders and the non-binders. Since the TPR and FPR are identical, this will result in a ROC curve that bisects the unit square Fig. (**2A**). In this case, the area under the ROC curve is simply one-half the unit square, or 0.5. Thus, a protocol that has no discriminatory power (equivalent to random selection), yields an AUC of 0.5. As discriminatory power improves, the binding affinity distributions and cumulative distribution functions are more widely separated. This shifts the ROC curve toward the upper left hand corner, and the AUC increases to a value between 0.5 and 1 Fig. (**2B** & **C**). When discrimination is perfect, the binders and non-binders are completely separated and the AUC value is 1. Significantly, it can be shown that the AUC value is identical to the probability that a randomly selected binder will be ranked ahead of a randomly selected non-binder, which is equivalent to the Wilcoxian statistic [51].

### Performance Variability

Conceptually, a null-hypothesis test can determine whether a docking protocol performs better than random in a statistically significant way. As a result of performance variability, a virtual screening protocol that performs randomly, on average, will also yield results that are better or worse than random. For example, Fig. (**3A**) shows the distribution of AUC values that result from a hypothetical randomly performing docking protocol. While, on average, the distribution is centered on an AUC of 0.5, performance variability results in much higher and much lower AUC values. For virtual screening purposes, it is desirable to create a method that on average performs better than random and to simultaneously assess the confidence of the results. Confidence can be quantified through probability, *i.e. *assuming the method performs randomly, what is the probability of observing an AUC value as large, or larger, than the value we estimated? The smaller the probability, the less likely it is that the value we observed was due to a randomly performing protocol. 

**Fig. (3):**
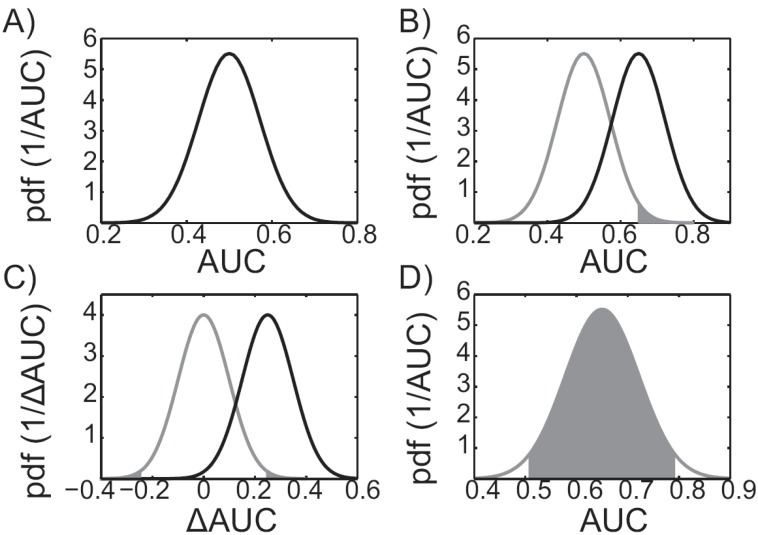
**Performance evaluation statistics. A**) Distribution of a virtual screening protocol that performs randomly, on average. **B**) An example p-value for a hypothetical virtual screening experiment with an AUC of 0.65 is illustrated as the shaded area under the null distribution, shown in grey. The alternative distribution, corresponding to the alternative hypothesis, is shown in black. **C**) The null distribution corresponding to the null hypothesis, “the two docking protocols perform identically,” is shown in grey. The p-value corresponding to two virtual screening experiments whose ΔAUC value is 0.25 is illustrated as the shaded areas under the grey null-distribution curve. The corresponding alternative distribution is shown in black. **D**) The 95% confidence interval of a hypothetical virtual screening protocol with an observed AUC value of 0.65. The 95% confidence interval is bounded by the grey shaded region, which contains 95% of the distribution.

In practice, a null hypothesis test can be carried out using the recipe outlined here. First, the null hypothesis: “the virtual screening protocol performs randomly” is assumed. The second step is to determine the corresponding null distribution, which can be determined using the central limit theorem and an analytic estimate of the standard error as in [45, 48, 49] (see, for example, Fig. (**3B**); note that the gray curve is the null distribution, centered on an AUC value of 0.5). In the third step, the null distribution is used to determine the so-called “p-value”, or the probability of observing an AUC value as large, or larger, than the value that was estimated (see the shaded region in Fig. **3B**). The fourth step is to consider the size of the p-value to decide whether the null hypothesis should be rejected in favor of an alternative hypothesis. If the p-value is smaller than some threshold, or significance level, we reject the null-hypothesis, replace it with an alternative hypothesis, and deem the result statistically significant. Popular significance levels for rejecting the null-hypothesis are 5% and 1% [52], though we note that these levels are arbitrary [53]. If the null hypothesis was rejected during the fourth step, the fifth step is to make the alternative hypothesis: “the protocol of interest performs better than random.” Subsequently, the alternative distribution must be determined, again using the central limit theorem and an analytic estimate of the standard error [45, 48, 49]. The average of the alternative distribution is typically taken as the AUC value of the virtual screening experiment (see, for example, the black curve in Fig. **3B**). The alternative distribution is important because it allows us to estimate confidence intervals. In the event that the null hypothesis was not rejected during the fourth step, one might consider alternative virtual screening protocols and repeat steps one through four until the null hypothesis can be rejected at the desired significance level.

If two virtual screening protocols perform identically, on average, then the distribution of AUC difference values (ΔAUC) will have an average of zero, as the gray curve in Fig. (**3C**) illustrates. To insure that the calculated ΔAUC is not due to the variability of two methods that perform identically (on average), a null hypothesis test must be carried out. This can be accomplished by following the recipe outlined in the previous paragraph, but assuming a different null hypothesis and distribution. The correct null hypothesis is: “the two different protocols perform identically.” The corresponding ΔAUC null distribution is centered on zero and can be estimated using the central limit theorem. Importantly, the standard error estimate must account for compound ranking correlation between methods [45, 48, 49]. Neglecting correlation may artificially inflate the standard error causing the null hypothesis to be inappropriately retained. The p-values are then determined as the shaded regions under the gray, null distribution, curve in Fig. (**3C**). The alternative distribution determined is shown in black in Fig. (**3C**).

Assessing how much performance variability to expect is necessary when considering a virtual screening protocol. For example, given an initial AUC value of 0.90, when a second database is screened, should an AUC value of 0.65 be surprising? Confidence intervals, a range of values that includes the true value with a given probability (confidence level), help address this question. The confidence level is typically taken at 95% [52], which implies that the true average AUC will be contained within identically constructed intervals in 95 of 100 identically performed virtual screens (with different databases). If we assume that the central limit theorem holds, then we can use the analytic estimates of the standard error to estimate the confidence intervals [53]. For example, Fig. (**3D**) illustrates a 95% confidence interval [54]. Typically, confidence intervals are estimated on the alternative distributions, discussed in the paragraphs above.

## GENERATING RECEPTOR CONFORMATION VARIABILITY WITH MOLECULAR DYNAMICS

Molecular docking, virtual screening and the other computational structure-based drug design methods discussed in previous sections have traditionally been performed on a single protein conformation. Despite this precedence, it is widely agreed that macromolecular structures have heterogeneity that cannot be represented by a single conformation, such as the crystallographic models deposited into the Protein Data Bank [55, 56]. In solution, proteins move and interact with their environment, and a crystallographic coordinate model can be represented as a network of physically interacting atoms by an equation and parameters called a force field. A force field allows us to calculate the potential energy of a system. Force field models are based on a variety of assumptions; most notably, the Born-Oppenheimer approximation, which allows us to describe the system solely as a function of nuclear coordinates. The simplified interactions are described by mathematical functions with few parameters, such as a harmonic potential and its corresponding spring constant. Making the physics simple allows parameters for the model, such as spring constants for bond vibrations, to be transferable to many different types of systems.

While much insight is gleaned from crystallographic models, many complex interactions and reactions require more dynamic detail. Molecular dynamics (MD) is a simulation method that propagates the system described by a potential function and numerical integration of Newton’s second law. Structural coordinates are propagated through time using fixed integration steps (typically 1 or 2 fs), according to the forces acting on the system. After each integration step, the forces on the atoms are recalculated and combined with the current velocity and positions resulting in new velocities and positions. The underlying physics of the fastest motions in the system, bond stretching, dictate a time step limitation of 1-2 fs. The positions calculated during a MD simulation are saved at periodic intervals generating a trajectory of snapshots in time, which approximate a statistical sample from an ensemble of configurations of the protein system at thermal equilibrium. Using this sample, time-averaged properties can be determined and connected to ensemble-averaged properties through the concept of ergodicity and the laws of statistical mechanics. While the accuracy of these averages depends heavily on proper equilibration and sampling of the system, qualitatively useful estimates can be determined in relatively short simulation times for small systems. 

Initially, one must decide on the type of force field to use when modeling a pharmaceutically relevant system. The energy models consistently used when examining atomic interactions are classical fixed-charge force fields such as AMBER, CHARMM, or OPLS [57-60]. These are typically used with one of several water models, such as TIP3P or SPC, and a generalized organic force field for ligand parameters, such as CGenFF or GAFF [61-63]. It is best to review the literature on the compatibility of water models with protein and generalized force fields being selected for use, as some energy functions were parameterized to be compatible only in specific circumstances [64, 65].

With most computational methods, a dangerous outcome is that results can be produced regardless of the expertise of the user. The fundamental learning curve is in setting up the system correctly. This entails taking into account relevant structures, force field parameters, protonation and ionization states, solvent and system sizes. A basic understanding of the underlying theory is critical for interpretation of the results produced. MD is essentially numerical statistical mechanics, therefore, users who have not been formally trained, should at least read some introductory text to familiarize themselves with such concepts before proceeding too far. Additionally, the highly technical nature of running and analyzing molecular dynamics simulations usually requires an apprenticeship in a laboratory specializing in these computational techniques. The choice of ionic strength and temperature of the simulation cell should be guided by relevant experimental biological conditions where possible, although current MD simulation standard practices do not come close to standard conditions in a cell [66]. With this construct established, the system is equilibrated until several metrics of stability are achieved. Structural stability is commonly measured by monitoring kinetic and potential energy time series, as well as two coordinate metrics, the root mean square deviation (RMSD) and root mean square fluctuation (RMSF). The RMSD is the coordinate position deviations averaged over particles at a fixed time point, and the RMSF is the variation in atomic position averaged over time. Typically, the RMSD is plotted as a function of time, and is a geometric measure of how different a protein conformation is from a reference conformation, while the RMSF is plotted on a per residue basis to describe local structural fluctuations. In some cases, particularly with proteins that undergo large structural rearrangements, many transitions between conformational macrostates will not occur, and the system will not reach equilibrium. However, meaningful information and structures near the starting conformation can still be extracted. Additionally, to improve conformational sampling local to a starting structure, multiple independent trajectories with different velocity distributions are often used [67], and the additional information can be integrated in the design process. 

Free software is available to run molecular dynamics simulations, such as the widely used Desmond, Gromacs, or NAMD packages [68-70]. Other commonly used dynamics packages, such as AMBER and CHARMM, are available at minimal cost to non-profit organizations [71, 72]. Many of these packages can be operated with limited experience, and have tutorials, user manuals and forums where users can have questions answered by developers and experts fairly rapidly. While there are numerous force field comparisons noted in the literature, there are limited direct comparisons of MD packages that are implemented for parallelization and scalability. While good packages read several force fields and the results should not depend on specific software implementation, often the processor number and type available to the user may dictate which package to use. 

Prior to using MD snapshots in SBDD pipelines, evaluation of the trajectories should be performed to assess the ensemble sampled. In particular, one question must be addressed when considering the use of MD snapshots in a drug design context: is the sampled phase space relevant to inhibiting the targeted mechanism? Phase space is a high dimensional concept representing all of the degrees of freedom of the system (i.e. for a system of N atoms, each atom can be described with 3 coordinates and 3 components of momentum, resulting in 6N phase space). One way to understand how a simulation is changing is by quantifying the phase space that is being sampled. For example, principal component analysis is a popular technique that decomposes the variance of atomic positions into principal modes [73]. Visualizing these modes is useful in identifying the collective motion responsible for large conformational events, such as binding pocket expansion. Alternatively, clustering and population analysis of a trajectory identify highly populated conformational regions of phase space and are other techniques that may be employed [24, 74]. By carefully analyzing the distribution of conformational states in the context of the project goals, useful insights can be identified. To help with such analyses, many tools freely available to visualize and analyze molecular dynamics trajectories exist, including, but certainly not limited to, the widely used VMD, Amber-Tools and Gromacs programs [69, 72, 75]. Open source tools for commonly used interpreted programming languages and packages such as Python and R are also available [76-79].

Currently routine time-dependent molecular simulations can access microsecond timescales, although it is more common to find studies publishing hundreds of nanoseconds of simulation [80]. Recent advances in software and hardware have thrust MD timescales forward. Some software has been implemented to run on graphics processing units (GPUs), which have more arithmetic logic units than conventional CPUs, but are not as complex. The clock speed is slower on the GPU but the access to memory is faster, and this, combined with GPU programming language development, has lead to potential for routinely achieving longer MD timescales. The promise of GPUs is further enhanced by massively parallel GPU hardware [81, 82]. Impressively, GPU hardware was recently used to sample a binding association event [83]. Previously, these time-scales were only accessible on a specialized hardware machine [83, 84]. 

## DEALING WITH BIG DATA: HOW TO GET THE MOST FROM DOCKING WITH MD

With ever improving hardware, molecular dynamics simulations will continue to be a ubiquitous biophysical technique, and rightly so: in the absence of multiple crystal structures, it is a good way to quickly and realistically generate conformational diversity. While other methods, such as normal mode analysis [85], can be used to generate conformational diversity, MD has the benefit of approximating the conformational ensemble expected at thermal equilibrium, which may facilitate virtual screening success. Contemporary MD simulations can easily result in tens of thousands to hundreds of thousands of receptor conformations, and a virtual screen can be performed against anyone of these [86], all of them [87, 88], or any subset [40, 41, 89]. Moreover, there are multiple ways to combine the docking scores from each receptor. As each choice of protein receptor(s) and score combination method can perform differently, a large hurdle in MD-based ensemble virtual screens is deciding which set of protein conformations to pair with which score combination method. In this section, we outline a set of best practices to systematically evaluate ensemble performance. 

Typically, the first step in performing a virtual screen using an MD trajectory is ensemble reduction. Ensemble docking scales linearly with the number of receptor conformations, and since only finite resources are available, culling a tractable conformational subset is essential. Several methods have been suggested in the literature, including RMSD-based clustering [44], QR factorization [40, 41] and binding-site volumetric clustering [90, 91]. While the RMSD-based clustering and QR factorization methods can be performed with freely available software, the binding-site volumetric clustering is currently implemented in commercial software. However, there are several freely available pocket-volume calculation programs [92, 93] whose output could be used to cluster in a similar fashion. With the variety of ensemble reduction techniques available, one may naturally wonder which performs best. Unfortunately, to the best of the authors’ knowledge, a set of conformational descriptors that correlate strongly to virtual screening performance has not been definitively determined [86]. Moreover, since the best ensemble reduction technique may be system dependent, methodically evaluating the performance of several techniques before performing a large-scale screen is a sensible approach, assuming available time and resources. With a set of structures in hand, an ensemble-based virtual screen can be carried out. Faced with designing an ensemble virtual screening protocol, which receptor conformations should be used? The simplest answer is to use them all. For example, given 20 receptor conformations and a database of 100 compounds, each compound would be docked into each of the 20 conformations, for a total of 2000 ligand scores. Then each ligand score can be a combined in some fashion yielding an ensemble score. While this simple approach has been used successfully in some cases [40, 41], it overlooks a combinatorial subtlety, and performance may suffer as a result. Alternatively, given 20 conformations, a smaller subset of *m* conformations can be chosen to constitute the ensemble [94]. The number of unique ensembles that can be constructed this way is given by the binomial coefficient. This can quickly result in a large number of possibilities to consider, each with a different performance. The number of unique ensembles increases as a function of size. For example, when ensembles are made from any of 20 unique receptor conformations, a maximum of 184,800 combinations are possible using 10-member ensembles Fig. (**4A**). The total number of possible ensembles that can be considered is determined by summing the number of unique ensembles that can be constructed at each ensemble size. For example, when ensembles are constructed from 20 unique receptor conformations, there are a total of 1,048,575 ensembles to consider. In practice, performance can be plotted as a function of size by extracting the top-performing member for each size [89, 94]. For example, Fig. (**4B**) plots the AUC of the top-performing ensemble as a function of ensemble size for a hypothetical vHTS experiment. While the data is contrived, the trend follows our own experiences and is similar to those that can be found in the literature [94]. Clearly, optimal performance occurs for a five-member ensemble, and this ensemble would then be considered against other top-performers, as described below. 

Once the top-performing ensemble has been identified, the process can be repeated using different score combination techniques. For example, one might consider arithmetically averaging ligand scores. Alternatively, ligand scores could be combined in a weighted average, where the weights may be extracted from clustering and population analysis [44], or may be taken as the Boltzmann weights [95]. Another popular method selects only the best ligand score for ranking purposes [94]. Additionally, as docking performance varies from one target to another [96], different docking programs should also be considered. Each method can then be compared using the simple statistics presented in the “Prediction and Performance Evaluation” section of this review to arrive at the optimal docking protocol for the system of interest. In principal, identifying the optimally performing ensemble method on a smaller validation database should increase the success of virtual screens performed on much larger databases. While this optimization scheme represents a significant resource investment, much of the analysis can be automated to reduce the required “hands on” time. Additionally, while this method is limited to targets with known actives, inexpensive semi-high through put techniques, such as differential scanning fluorimetry [97], coupled with free databases, like the National Cancer Institutes Diversity set, may facilitate validation database development [98].

## CONCLUSIONS

Molecular dynamics is increasingly being included in academic high-throughput efforts for pharmaceutically relevant prediction research, and the future is bright. For example, using SciFinder Scholar to track citations involving molecular dynamics research, one can see in Fig. (**5**) that molecular dynamics simulations carried out with respect to drug design has been exponentially increasing since the 1980s, and has been increasing in the context of virtual screening and binding site prediction since the early 2000s [99]. While a gold standard practice for selection of MD snapshots for virtual screening and binding site prediction has yet to be established, the growing number of academic research laboratories carefully considering this as part of their discovery toolkit improves future prospects of structure-based drug design. Similar optimism can be found in related reviews discussing methods of incorporating receptor flexibility into the drug design process [2, 81, 100, 101]. Molecular dynamics is becoming an increasingly practical tool to enhance drug discovery and design efforts. The performance of hardware and software is continuing to improve, and a growing number of researchers are turning their minds toward tackling the most pressing challenges facing the field. The authors’ are optimistic that these factors will synergize to make the successful application of molecular dynamics simulations to structure based drug discovery and design problems routine. 

## Figures and Tables

**Fig. (1):**
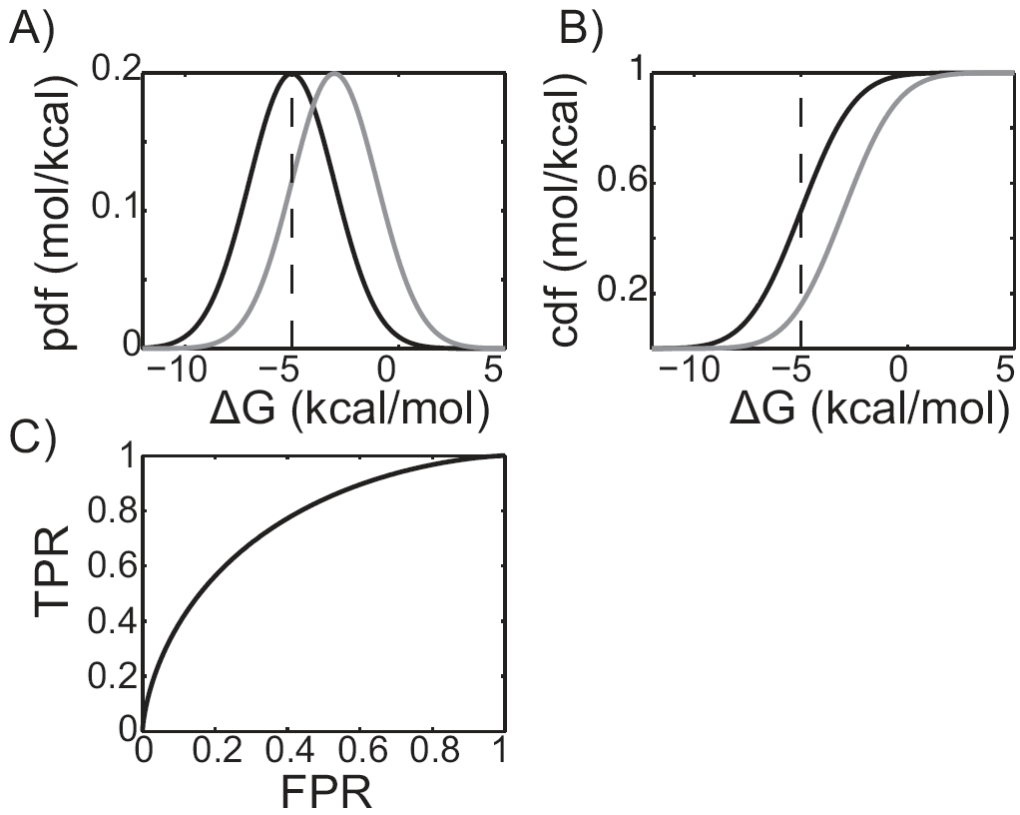
**Receiver Operating Characteristic (ROC) plots. A**) Predicted binding affinity probability distribution functions (pdfs) of the actives, shown in black, and inactives, shown in grey, for a hypothetical virtual screening experiment. A -5 kcal/mol threshold is shown as a grey, vertical dashed line. Compounds whose scores lie to the left of the line are experimentally assayed. Compounds whose scores lie to the right are not. **B**) The integrals of the pdfs, or cumulative distribution functions (cdfs). The black cdf is equivalent to the true positive rate (TPR), while the grey cdf is equivalent to the false positive rate (FPR). **C**) A ROC plot, generated by plotting the gray cdf along the X-axis versus the black cdf along the Y-axis.

**Fig. (2):**
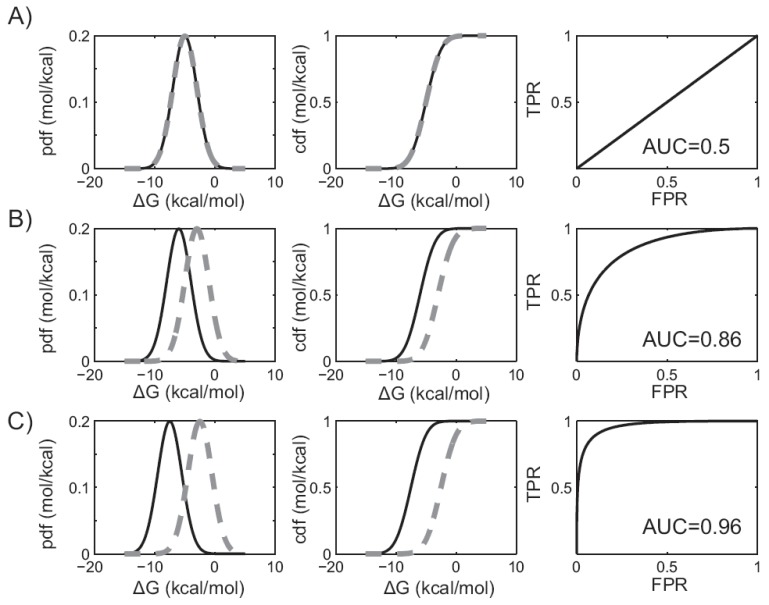
**The area under the ROC curve and virtual screening performance.** In figures **A**) through **C**), plots in the leftmost column describe the predicted binding affinity probability distributions. Plots in the middle column give the corresponding cumulative distribution functions. The binders are shown in solid black, while the non-binders are shown in dashed grey. Plots in the right column show the corresponding ROC curves. AUC values are shown for three different hypothetical virtual screening protocols with **A**) no discriminatory power, **B**) improved discriminatory power, or **C**) near perfect discrimination.

**Fig. (4):**
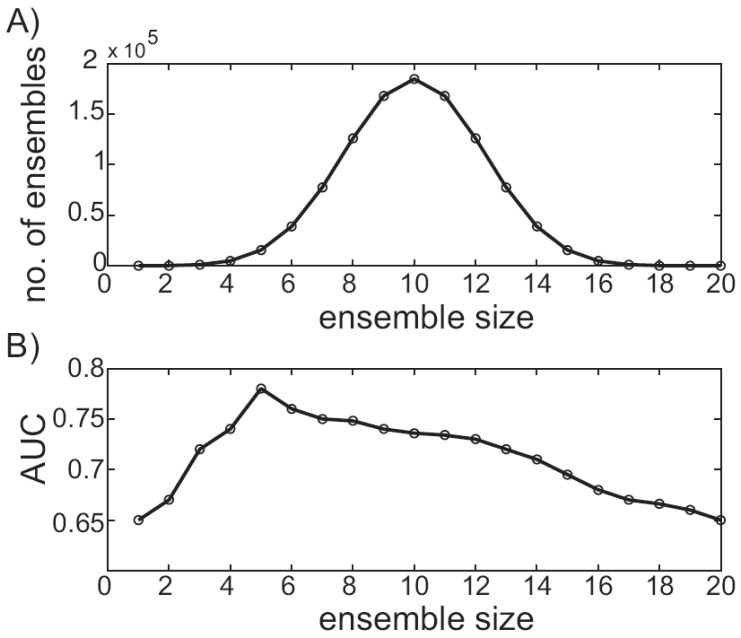
**Ensemble size and virtual screening performance variability. A**) For 20 receptor conformations, the number of ensembles that can be constructed is plotted as a function of the ensemble size. **B**) For a hypothetical ensemble-based virtual screening experiment, the AUC of the top-performing ensemble is plotted as a function of ensemble size.

**Fig. (5):**
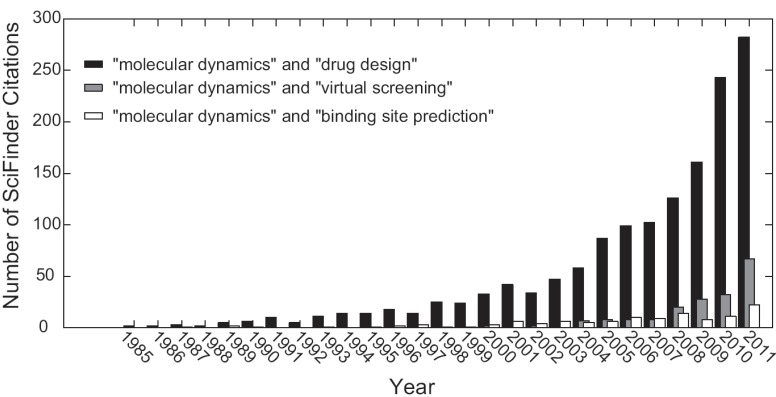
**Exponential increase in molecular dynamics citations.** Searches using the quoted keywords were performed using SciFinder Scholar. The number of citations returned is plotted as a function of year, indicating the increasing use of molecular dynamics in drug discovery.
